# Overcoming institutionalised barriers to digital health systems: an autoethnographic case study of the judicialization of a digital health tool

**DOI:** 10.1186/s12911-022-01769-x

**Published:** 2022-01-31

**Authors:** Dudzai Mureyi

**Affiliations:** Department of Biomedical Informatics and Biomedical Engineering, Faculty of Medicine and Health Sciences, The University of Zimbabwe, Harare, Zimbabwe

**Keywords:** Digital health, Institutional theory, Barriers, Zimbabwe, Law, Autoethnography

## Abstract

**Background:**

The deployment of digital health systems may be impeded by barriers that are, or are linked to underlying enduring institutions. Attempting to challenge the barriers without addressing the underpinning institution may be ineffective. This study reflects on ways actors may surmount institutionalised barriers to the uptake of digital tools in health systems.

**Methods:**

I applied Institutional theory concepts to an autoethnographic case study of efforts to introduce a digital tool to provide citizens with medicines information.

**Results:**

The tool’s uptake was impeded because of state regulators’ institutionalised interpretation of pharmaceutical advertising laws, which rendered the tool illegal. I, along with allies beyond the health sector, successfully challenged the regulators’ institutionalised interpretation of pharmaceutical advertising laws through various actions. These actions included: framing the tool as legal and constitutional, litigation, and redefining these concepts: ‘advertising’, ‘health institution’, and the role of regulatory bodies vis a vis innovation.

**Conclusion:**

After identifying a barrier as being institutionalised or linked to an institution, actors might challenge such barriers by engaging in institutional work; i.e. deliberate efforts to challenge the relevant institution (e.g. a law, norm or shared belief). Institutional work may require the actions of multiple actors within and beyond the health sector, including judicial actors. Such cross-sectoral alliances are efficacious because they provide institutional workers with a broader range of strategies, framings, concepts and forums with which to challenge institutionalised barriers. However, actors beyond the health system (e.g. the judiciary) must be inquisitive about the potential implications of the digital health interventions they champion. This case justifies recent calls for more deliberate explorations within global health scholarships and practice, of synergies between law and health.

## Background

Digital systems hold promise for healthcare delivery and attract billions in investment worldwide [1]. Their deployment however, is complex and vulnerable to barriers [2, 3]. Such barriers can be technological, contextual or human-related [2]. Technological factors are related to the information system’s inherent features such as connectivity or the usability of its in-built components. Contextual factors have more to do with the social environment within which the information system is introduced while human factors are related of the system’s intended users. Barriers related to contextual and human factors may be underpinned by institutions or may be institutions themselves. (Institutions being practices, customs, ideas, beliefs, shared understandings, objects etc., that shape social interactions, have attained the status of ‘taken-for-granted facts’ [4], tend to be invisible [5] and resistant to change [6]. This premise is the basis for this paper, which considers institutionalised barriers to the deployment of digital health systems and some of the ways those barriers can be overcome. It is worth remarking that although in this paper, I consider institutions from the perspective of them being linked to implementation barriers, institutions, like many factors in digital health projects, are not inherently barriers. Institutions can be implementation barriers or implementation facilitators, depending on context [7]. In this case study, institutions that aided the implementation of the digital health tool being considered may well have existed. However, it was the impeding institution that more readily recommended itself to empirical study because of its conspicuousness.

Institutions may be considered a source of stability, order and resilience [8, p. 57, 9]. Therefore healthcare, being characterized by well-established and enforced rules and routines, is a particularly institutionalized field [10]. This tends to complicate the implementation of digital health information system projects because such projects may require substantive changes to established routines [11]. Indeed, some digital health information system implementation failures are explained by “resistance to change” [7, 12]. Examples of digital health system projects that were impeded by barriers linked to institutions exist. I have listed three of these below. In these examples, actors have responded to institutionalised barriers in various ways. Some abandoned the implementation of the project altogether. Others developed parallel, paper-based workarounds to complete tasks instead of confronting the relevant institutions. Others still, tried to address the barrier by only communicating the benefits of the technological innovation, without pursuing targeted ways to address the underlying institutions.

1. Plans for a transparent pharmacy information system were abandoned despite the benefits being communicated and understood by stakeholders. Plans were abandoned because stakeholders were united in their opposition to those plans based on their *shared understanding* of competition and the proprietary nature of pharmacy records [13].
2. Implementation of a computerised prescribing system was discontinued because of physicians’ belief in the professional *norm* of spending face-time with patients. Face-time was compromised when the physician spent time working the computers [14].
3. The perceived illegality of electronic prescriptions of medicines resulted in their rejection by pharmacies that feared *regulatory reprimands.* Physicians then used paper-based workarounds instead. This rejection was despite sustained communication to pharmacies about the convenience and legality of e-prescribing systems [15].

Given that barriers to the successful deployment of potentially beneficial digital health systems can be underpinned by institutions, which in turn tend to be enduring, how can actors overcome institutionalised barriers to digital health systems? This is the question that I address in this paper, using two lenses from institutional theory explained immediately hereafter: “Institutional work” and “institutional logics”. These lenses together, offer concepts that define, identify and illuminate:

- institutions,
- actions that challenge institutions and
- the structural rule systems which shape what kind of actions are legitimate and possible.

## Theoretical framework

### Institutions

Institutions endure because they are supported by at least one of three ‘pillars’: the regulative pillar, the normative pillar and the cultural-cognitive pillar [8, pp. 59–70]

- The regulative pillar: a system of formal laws and entities that are empowered to punish noncompliance with the institution concerned.
- The normative pillar: a system of values and informal rules that shape expectations and dictate what’s considered acceptable behaviour. The normative pillar supports institutions that are or are based on societal (including professional) values or norms.
- The cultural-cognitive pillar: a system of shared understandings, beliefs or ways of making decisions in given situations. This pillar upholds institutions by rendering alternatives inconceivable or unsound [8, pp. 59–70].

In other words, the regulative pillar dictates ‘what must happen”, the normative pillar is about ‘what should happen’ and the cultural-cognitive pillar is about ‘what generally happens’ [9].

### Institutional work

According to Lawrence and Suddaby [16], institutional work is “the purposive action of individuals and organizations aimed at creating, maintaining and disrupting institutions”. Actors tend to disrupt institutions by engaging in three types of institutional work: *disconnecting sanctions and rewards, dismantling moral/normative foundations and undermining assumptions and beliefs*. These forms of institutional work are often done by redefining, recategorizing, reconfiguring, and problematizing social phenomena and concepts [16].

*Disconnecting sanctions and rewards* is coercive and can be useful for targeting an institution’s regulative pillar. It typically involves actors using state apparatus such as the judiciary, to invalidate or nullify certain laws or taken-for granted phenomena. Without state intervention, these laws or taken-for granted phenomena would endure because actors are coerced into upholding them through sanctions or rewards. Apart from having the power to invalidate institutions, the state also has the power to set new standards or new technical definitions or meanings and actors may invoke this power to replace previously taken-for-granted standards, technical definitions or meanings [16]. A critical aspect of the institutional work that disrupts institutions thus involves *defining and re-defining* ideas and concepts in ways that change what actors are permitted to do [17]. Actors that possess the financial or intellectual resources to enlist the state’s judiciary to achieve their goals are the most likely to engage in this kind of institutional work [16].

*Dismantling moral/normative foundations* tends to target the normative pillar. Rather than a direct attack on the target institution, as is the case when state apparatuses are used to carry out institutional work, this kind is gradual and indirect. It involves *problematizing or questioning what has long been considered normal, right, appropriate or acceptable*. Actors most likely to successfully undermine the moral/normative basis of institutions are elites in their fields [17]. Their prestige capacitates them to develop and articulate their justifications for reimagining what is *appropriate*. Other actors can thereafter adopt the newly-emerged trends [16].

*Undermining assumptions and beliefs* targets the cultural-cognitive pillar. It undermines taken-for-granted assumptions and beliefs about how things can be done. Institutional work here involves gradually undermining an institutionalised practice by engaging in contrary/new practices [16]. It can involve inventing and communicating new ways of doing things that challenge institutionalised beliefs and assumptions about what is or isn’t *conceivable or possible.*

### Institutional logics

Although institutional work is conducted by purposive actors with agency, their agency is not unlimited. Rather, it is delimited by rule systems (institutional logics) that enable or preclude certain actions [16]. An institutional logic is ‘how a particular social world works’ [18]. It is a system of rules and principles that provide general guidelines for how occupants of a particular sphere of society ought to conduct social interactions if they are to be considered rational and legitimate in that particular sphere. Society comprises several social spheres (e.g. the marketplace, the professions domain, the social world governed by the state) [19], each with a central logic. Actors may be influenced by more than one logic and they respond differently to this complexity; choosing which logic to conform to, which one to ignore, when and how to conform and ignore. This co-existence of multiple institutional logics (e.g. in a health system) can be a resource available to actors to leverage as they carry out institutional work because they provide multiple ways for actors to legitimise their actions [20].

I have summarised the three institutional logics and their characteristics that are relevant in this paper in Table 1 [21]. They are as follows:

**Table 1:** Characteristics of the professional, state technical and judicial logics that are most relevant to the discussion in this paper [21]

	Professional logic	State technical logic	State judicial logic
Guiding values	Patient interest	Public interest	Constitutionalism
Actors with the legitimacy to debate issues that concern healthcare interventions	Regulators of professions Healthcare professionals and their representatives	Regulators of professions Healthcare professionals and their representatives	Legal practitioners, Litigants (any rights-bearing individual)
Decision-making forums	Stakeholder meetings and other groupings of health professionals Regulators’ internal committee meetings	Regulators’ internal committee meetings	Courts of law
Decision-makers with coercive power	Healthcare practice regulators	Healthcare practice regulators	Judges

The *professional logic*: defined generally by its privileging of patients’ best interests, the recognition of regulators as the legitimate decision-makers over healthcare practice and the recognition of meetings of professionals as legitimate forums for decision-making.

The *state technical logic*: characterised by public interest, the recognition of state regulators as the legitimate decision-makers and the internal committee meetings of government entities, (e.g. regulators) as forums for decision making.

The state judicial logic is characterised by a concern with constitutionalism, courts as decision-making forums, legal practitioners as legitimate actors and judges as decision makers in pharmacy matters. ‘Access to health’ issues (and most issues) are regarded as constitutional matters. Table 1 is not meant to imply that other logics do not exist in the research context. I have only described the logics that are identifiable in the data analysed in this paper.

**Fig. 1:**
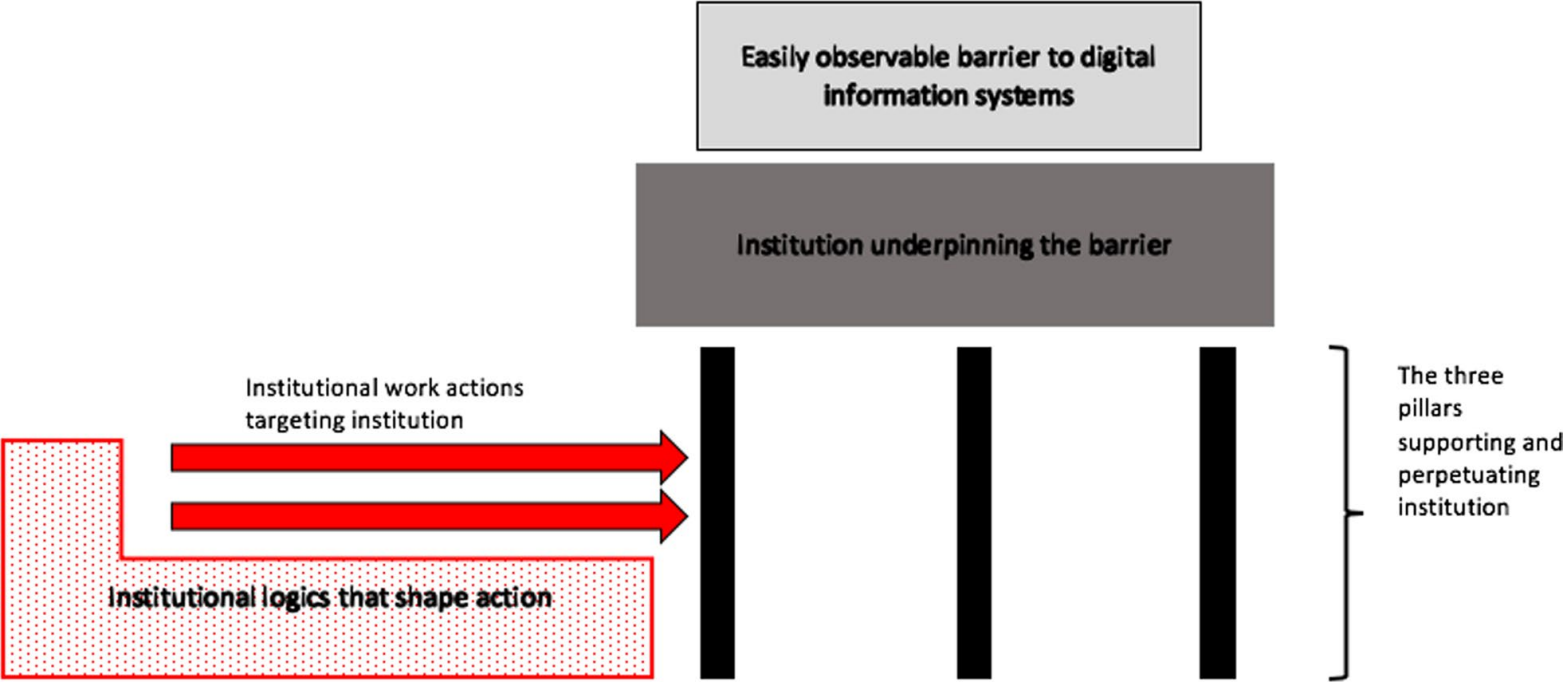
Relationship between barriers to digital information systems, institutions, institutional work and logics

Figure 1 depicts the relationship between barriers, institutions, institutional work and institutional logics that has just been described.

To summarise, institutional work focuses on actions, is concerned with the purposive, goal-oriented actors who possess agency, and pays attention to the relationship between that agency and the underlying structural rules that encourages or discourages particular actions.

## Case description

Zimbabwe’s well-documented economic and health system challenges [22–24] included medicine shortages. The impacts of shortages were compounded by well-intentioned Direct-To-Consumer Pharmaceutical Advertising (DTCPA) regulations. These regulations prohibited pharmacies from broadcasting details of their medication inventory or prices to the general public. In the wake of shortages, pharmacies that had particular medicines absent at other pharmacies, couldn’t broadcast this fact. Therefore, patients needing medication relied on door-to-door enquiries at pharmacies [25].

**Fig. 2:**
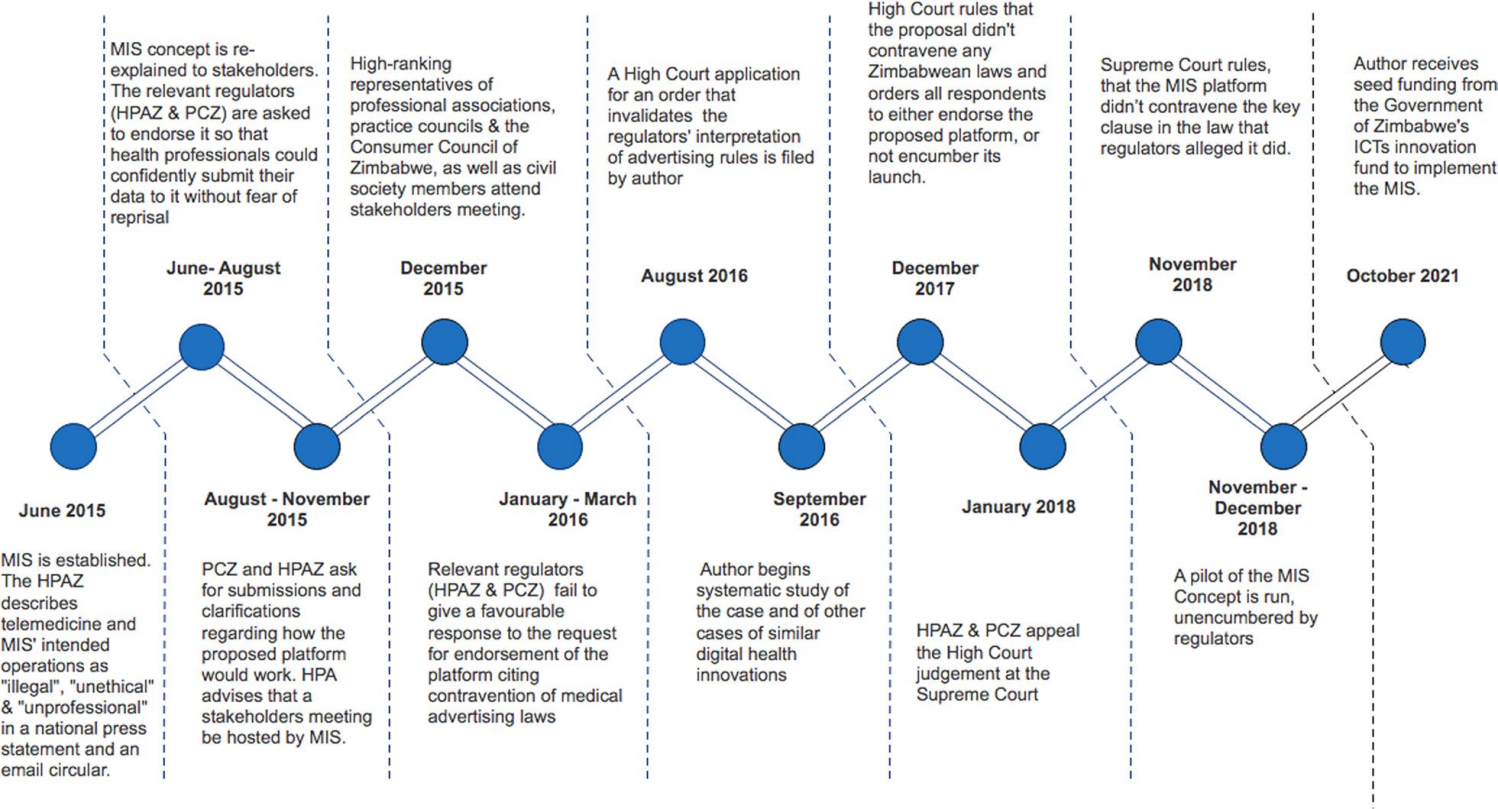
Case event sequence

In 2015, as a pharmacist in Zimbabwe, I established ‘The Medical Information Service’ (MIS), a social enterprise intended to maintain an online database, of pharmacies in Zimbabwe with the following details for each: their physical address, contact details, which payment methods they accepted, their opening hours, and which medicines and devices each had in stock at any given moment. The system would then respond to queries submitted by any person via an internet-enabled communication device. Enquirers would be given provider information based on their geographical location, their method of payment, and the medicine being looked for and the time of day of the search. MIS was met unfavourably by the relevant regulators. They considered this concept to be a violation of the advertising provisions in section 135 of the Zimbabwean Health Professions Act, which prohibits direct-to-consumer advertising of medicines and health services. The state regulators’ view that MIS violated advertising provisions was due to an interpretation of medical advertising laws that was ultimately overturned in the court of law (see Fig. 2 for the events timeline). The state regulators concerned were: The Health Professions Authority of Zimbabwe (HPAZ) and the Pharmacists Council of Zimbabwe (PCZ).

## Methods

### Study design

I conducted a qualitative case study which employed documentary analysis and autoethnographic techniques. Autoethnography involves the analysis of autobiographical experience. Results of such analyses then help to illuminate broader sociocultural phenomena. Different autoethnography approaches exist. They differ based on narration style and the extent to which the author is the researched phenomenon. Autoethnographies can be ‘evocative’—these are descriptive personal stories, positioning the author as the main object of narration. They attract the most criticism from traditional social scientists [26, 27]. This work is not an ‘evocative’ autoethnography. ‘Analytical’ autoethnographies by contrast, demonstrate a commitment to theoretical exposition, engage with data sources beyond the self, and show analytical reflexivity [27]. Often, as is the case in this instance, analytical autoethnographies only include the researcher’s experiences and texts as data, to the extent to which this cannot be avoided without impoverishing the analysis [26]. Forms of autoethnography differ in how much emphasis is placed on the study of others, the researcher's self and interaction with others, traditional analysis, and the interview context, as well as on power relationships. A detailed exposition the different forms was amply done by Ellis, Adams and Bochner [26]. This paper, takes the narrative autoethnography form. It refers to texts that infuse my experiences with the ethnographic descriptions and analysis of others. Here the emphasis is not the study of the author. Research goals are achieved partly by attending to encounters between the narrator (myself) and members of the groups being studied and the analyses of processes [26]. I made both the decision to scientifically analyse the data, and the methodological choice to draw from autoethnographic techniques for analysis, in 2020.

While autoethnographies have some advantages, e.g. they avail privileged data that’s inaccessible to other researchers [28], like any research method, autoethnography has limitations. It places researchers at risk of producing descriptive inward-looking accounts at the expense of theoretical insights. This is mitigated by extensive peer-review [29], limiting the reliance on autoethnographer’s memory for data, triangulation and linking data with theory [30]. Therefore, to achieve acceptable levels of validity in this autoethnography:

- documented evidence was privileged over my own memory (which cannot be audited)
- autoethnographic data was triangulated with insights from 48 formal in-depth interviews and Zimbabwean pharmacists’ online communities of practice [21]. Some of these 48 interviewees were members of the decision-making committees at HPAZ, PCZ and the national medicines regulatory authority. Furthermore informal conversations with members of the decision-making committees at HPAZ, PCZ also helped to clarify the context within which some of the formal documents analysed in this paper, were written.
- I privileged critical analysis (using concepts from institutional theory), over descriptive narration.
- the contents of this paper, authored in the context of doctoral work, were peer-review [29] by five academics from the Universities of Edinburgh, Manchester and Oxford, and a board-level officer of both regulatory organizations that I mention in this paper.

Various definitions of ‘case study’ exist. For the purposes of this paper, I rely on Merriam’s [31] definition of a qualitative case study as “an intensive, holistic description and analysis of a single entity, phenomenon or social unit. Case studies are particularistic, descriptive, and heuristic and rely heavily on inductive reasoning in handling multiple data sources” (p. 16). The utility of case study research is especially apparent when the phenomenon to be studied is rare or cannot be investigated experimentally or when the practical realities of resource constraints render the study of multiple cases unfeasible [32]. All these conditions applied to this research. The case study approach has been criticised for its particularism which is claimed to limit the generalisability of its findings [33]. Such criticism is considered by case study researchers to be based on the inappropriate application of statistical concepts to case study research [34, 35]. The many sub-units within a case make it possible to conduct several within-case analyses. Triangulation is also made possible through the use of diverse data subjected to interrogation by several methods, from various perspectives. For instance, the data for this paper’s case study comprised: 53 naturally occurring documents and descriptions of my experiences.

Documents presented several advantages [36] as data sources for an autoethnographic case study. I obtained them through inobtrusive methods. They were available to me already in transcribed form and I had easier access to them by virtue of my proximity to the case. Documents allowed me access to data co-produced by many authors that are typically not readily accessible for interviews (e.g. lawyers, judges, regulatory committees). Moreover, documents, dated at the time of production, contained stable content that was not vulnerable to the effects of my own recall bias. The major limitation of documents as data was the limited information they provided [36], especially regarding their implicit meanings [37] or the context at the time they were written. I managed this limitation by analysing multiple documents and sections of documents that expressed similar sentiments, identifying patterns as well as conducting formal and informal interviews with actors that had been privy to the internal context at regulatory institutions around the time some of the documents were created.

### Data collection

Data relevant to the case was available as documents accumulated since June 2015. All (N = 53) documents which I had access to, and directly pertaining to the case, were added to the dataset without applying inclusion criteria, during the month of May 2020. I kept them in a cloud-based location which my research supervisors also had access to. I digitised paper copies and labelled all documents by date [YYYYMMDD) so that analysis could be chronological. The documents that I analysed were: my letters to and from public institutions and officials, press and email circulars from regulatory institutions regarding digital health, newspaper articles about the MIS, stakeholders’ meeting minutes and court documents (affidavits, arguments and judgements) regarding MIS, and the legislative instruments that govern health professions in Zimbabwe. I also drew from supplementary data in the form of interviews that I conducted with Zimbabwean pharmacists. Ethics approval for the interviews was granted by the Joint Research Ethics Committee for University of Zimbabwe College of Health Sciences and the Parirenyatwa Group of Hospitals (Ref: JREC 295/18), as well as the University of Edinburgh’s Usher Research Ethics Group (UREG). Private correspondence that constituted part of the data quoted in this paper became public following the institution of court proceedings. These letters can be accessed by any person by inspecting court records in Zimbabwe where Court records are public records. The High court and Supreme Court case numbers are: HC8602/16 and SC12/18 respectively. For readers unable to access Zimbabwean court records, the full corpus of documents is available from me on reasonable request. One of the quotes that I cited in this paper was obtained from a social media platform (LinkedIn). I sought and obtained consent from the author of this post.

I accorded special salience to documents that directly referenced the advertising laws codified in Zimbabwe’s Health Professions Act i.e. applications for endorsement from me to the regulators, decision letters from regulators regarding MIS, minutes of the stakeholders meeting I hosted, submissions made to practitioners, pieces of legislation and court documents. I privileged these documents because they referenced the institutions of interest in this study (advertising laws and the interpretations thereof).

Consistent with the autoethnographic method, data also included my own reflections and details about my experiences as a researcher grappling with autoethnography as method, and my experiences as an innovator advocating for the acceptance of the MIS. To increase the trustworthiness of this type of data, I provide supporting quotes from traceable data sources (documents, social media posts, or interview transcripts).

#### Analysis

I reviewed research that has examined institutional work, for guidance on how to conduct similar analyses [20, 38]. For this paper, I subsequently followed these steps:

1. Ascertaining whether the institution involved was indeed an institution by examining the identified institution against the diagnostic characteristics of institutions
2. Outlining events of the case chronologically, identifying ‘who did what, when’ and ‘who said what, when’
3. Open coding of the data [32] to identify distinct actions that were purposive and directed at the institution in question
4. Identifying the different institutional logics that make the different institutional work actions possible by examining each action against the organizing principles of the different logics in the context.
5. Axial coding [39], to group actions thematically based on whether they undermined the beliefs and assumptions, targeted the moral or normative basis of the institution or used state organs, to directly invalidate an institution This was a deductive process based on Lawrence and Suddaby’s framework on institutional work [16].
6. Identifying the implications of the findings

The process steps 3–5 are depicted in Fig. 4.

I made the choice to apply analytical frameworks from institutional theory during the substantive analysis stage for two reasons. First, institutional theory comprises a set of well-developed lenses capable of explaining a wide range of actor behaviours [9] that I observed during the substantive analysis of this case study. Second, I conducted this work under the guidance of a doctoral supervisor whose areas expertise included institutional theory. I therefore benefited from guidance, peer review and support, to ensure that the quality of analysis was sound.

## Results

The results of analysis will now be presented in three subsections. In the first section, I solidify the assertion that the interpretation of advertising rules that rendered the MIS unacceptable to regulators was indeed an institution. (Before demonstrating how institutional work occurred in the case under review, it is important to confirm the premise that an institution that needed to be challenged, actually existed). In the second and third sections I describe the institutional work carried out by (a) a single actor and (b) multiple actors from beyond the health sector. The institutional work observed in the case included:

- compliance with the dominant actors’ demands,
- communicating and explaining new ideas to decision-makers,
- problematising the status quo,
- (re)defining concepts and ideas in ways that make new technological interventions acceptable and
- enlisting the help of allies even from beyond the health sector who possess coercive judicial power.

### Evidence that the inhibiting interpretation of advertising laws was institutionalised

To facilitate providers’ uptake of the intervention proposed by MIS, explicit and public endorsement or approval from the state was important. The approval was important to the extent that it signalled to practitioners that it wouldn’t be illegal to participate in MIS’ intervention. This endorsement was however withheld, and this withholding of approval or endorsement became a barrier to uptake. The reasons provided for the withholding of endorsement or approval suggested that an inhibiting interpretation of advertising laws, was the underlying basis for this barrier.

the committee carefully considered all the documents at hand and resolved to advise that your proposal is not in line with the Health Professions Act…in particular, Section 135. Furthermore, your proposal is not in line with the operations of a health institution based on the interpretation of the definition of a health institution as outlined in the provisions of Section 2(1)(a)(b) and (c) of the Health Professions Act **[20160301_PCZ_Decision_letter]**

it was also argued that,

it would not be within public interest to give positive response to the issue of the MIS as there are no satisfactory mechanisms to protect the public **[20160331_HPAZ_Decision_letter]**

Although regulators objected to the MIS for several reasons apart from its supposed contravening of advertising laws, I focus mainly on the interpretation of advertising laws as the institutionalised barrier that impeded the acceptance of MIS. This is because that interpretation was what exhibited the status of being institutionalised. It was also the point of greatest contention during litigation.

**Fig. 3:**
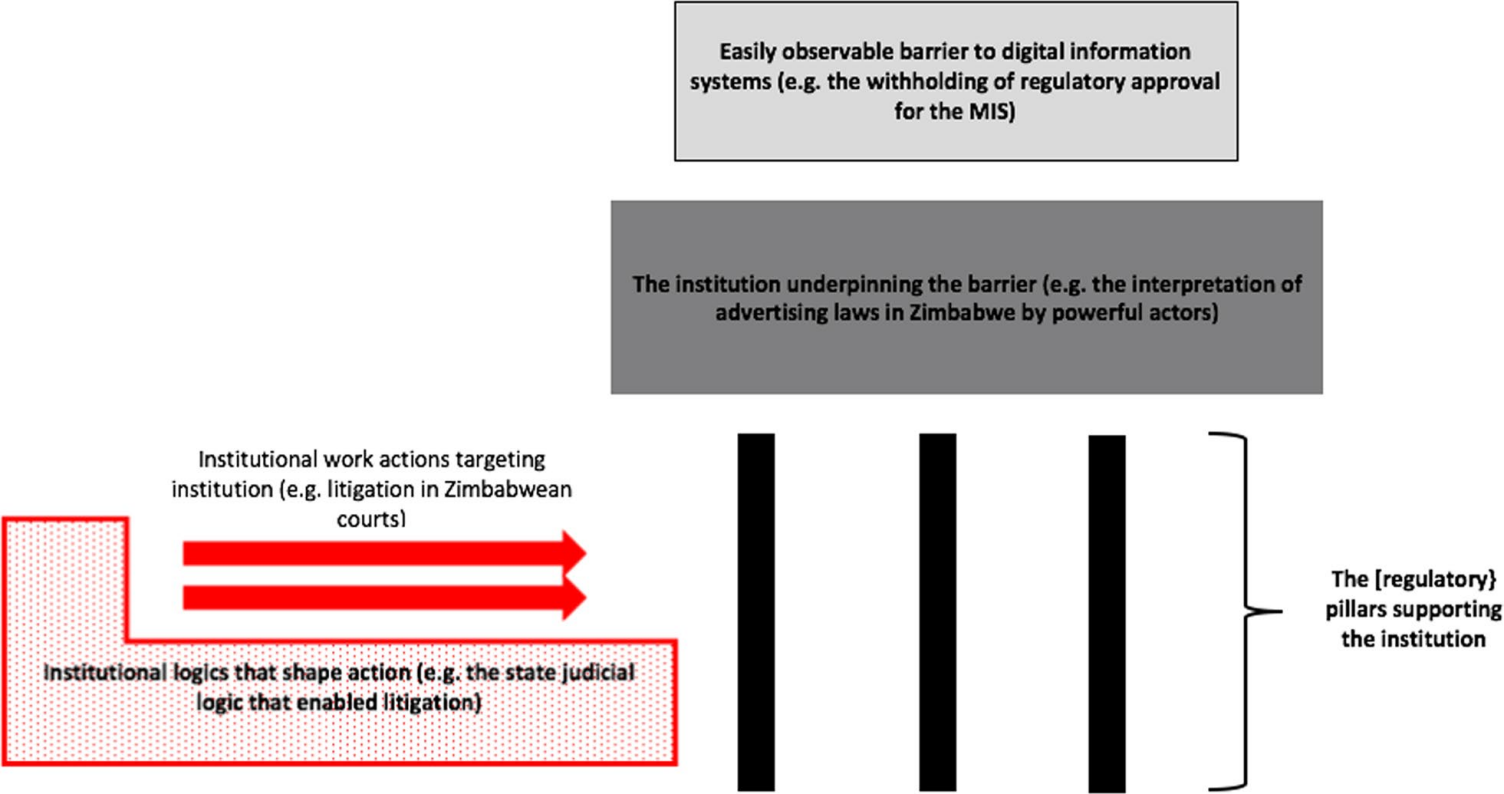
Relationship between barriers to digital information systems, institutions, institutional work and logics

**Fig. 4:**
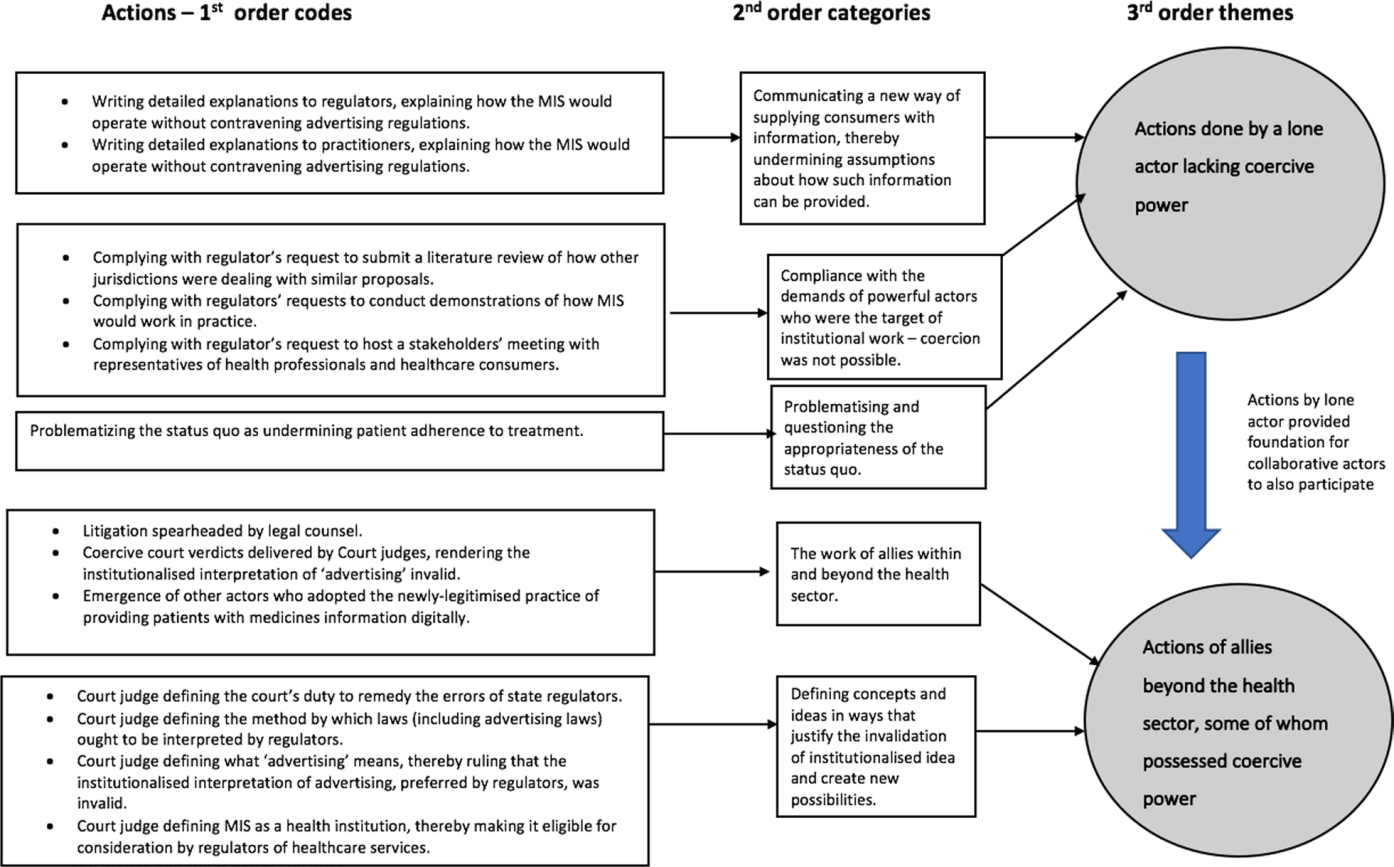
Process of data analysis

This interpretation of advertising laws met several diagnostic criteria for institutions:

- It resisted change for three years (2015–2018) despite several attempts to explain an alternative interpretation to the regulators.
- It was consistently communicated in official correspondence as taken-for-granted fact despite it being later invalidated by two courts of law,
- it was supported by at least one of three pillars that underpin institutions, i.e. the regulative pillar (a legal framework and regulators with the mandate to punish noncompliant actors) (see Fig. 3, derived from Fig. 1, shown here with specific examples from the MIS case) (Fig. 4).

### Institutional work by a lone actor

The three types of actions that I consider here involved:

1. Compliance with the demands of powerful actors (i.e. regulators). I hoped that by complying with regulators’ demands for: more information, wide stakeholder consultation and practical demonstrations, I would abate their objections to the MIS.
2. The production of texts that communicated to regulators and practitioners about how the proposal to operate the MIS did not constitute illegal advertising of medicines or health services. I envisaged that if they understood that what MIS proposed to do was not illegal, regulators would endorse it. Furthermore, in communication, I emphasised how allowing the MIS to operate was the constitutionally appropriate thing to do, given that the rights to information and to healthcare, were provided for in Zimbabwe’s Constitution.
3. The production of texts that problematised and questioned the appropriateness of the status quo for patient health.

My decisions to pursue these three actions were shaped by the professional logic and the state technical logic, which prioritise patient interests and recognise regulatory institutions as the legitimate gatekeepers of the healthcare institutional field in Zimbabwe, whose approval ought to be secured before the deployment of a healthcare intervention. My decision to frame what the MIS intended to do as legal and constitutional was influenced by the state judicial logic which views matters from the perspective of legality and constitutionalism.

1. Compliance with the demands of powerful actors (i.e. regulators)

From the time I introduced the MIS concept to regulatory bodies for endorsement, several requests for more information and stakeholder consultation were made by the regulators, before they ultimately communicated the decision that the MIS could not be legally operationalised. For example:

MIS is requested to organise and fund a stakeholders’ demonstration workshop where you would make a presentation to the stakeholders in the medical industry. During the presentation, stakeholders will ask questions, give fair input and get a better understanding of your proposed project **[20151021_HPAZ Request_for_Stakeholders_Meeting].**

I complied with each request, including the request to host a stakeholders’ meeting at my own expense. Although the stakeholders’ meeting lacked coercive power to make binding enforceable decisions, it was a legitimate decision-making forum according to the professional logic. It was therefore hoped that any endorsements coming from the stakeholders would persuade the regulators to shift their position. I approached each request and meeting with considerable optimism, resting in the belief that when presented with explanations that would align with their cognitive models, regulators and other stakeholders would endorse the MIS. I believed that their apprehension was merely because I was not communicating the mechanical workings of the innovation effectively. This kind of [naïve] optimism, is a trait consistent with many entrepreneurs at the varying stages of their journey [40]. Later, in the High Court judgement, the judge would refer to my apparent optimism:

[Mureyi]’s proposal letter contained reasons why she felt that her application would not be met with contention…she reasonably felt that the [MIS] database would not be problematic… **[20171213_High_Court_Judgement]**

It is my view that on its own, compliance with these requests was ineffective in changing regulators’ perception around what counted as illegal advertising, and ultimately, perceptions around the legality of the MIS.

I have done my best to persuade 2nd respondent {PCZ] to approve my idea without success. **[20160824_My Founding Affidavit to High Court]**

This highlights the challenges encountered by actors seeking to disrupt institutions in the sector within which they are embedded. Actors within an institutional field (in this case, professionals within the health sector) may find it challenging to disrupt institutions within their field because as they try to do that, they are limited by that field’s dominant arrangements or beliefs that shape even their own cognitions and decisions [39]. These actors try to institute change while acting within the limiting confines of the logics that influence both them and the actors they wish to change. In this instance, the professions logic belief held by advocates of MIS, that healthcare regulators were important actors whose demands needed to be acquiesced to in order to convince them to embrace an intervention, was actually a hinderance for a year (see case timeline).

2. Production of texts that communicated and explained to regulators and practitioners how the MIS’ intended operations did not constitute illegal advertising of medicines

The first indicator that advertising rules were being interpreted in an inhibiting way was a global email communication sent to all healthcare providers in Zimbabwe by the HPAZ warning them that getting involved with the me and the MIS “would be advertising, which is prohibited by the Health Professions Act. This had the obvious result of deterring healthcare professionals from participating in the operationalisation of the MIS.

I have approached the Respondents with my idea. I required their approval. Their approval is a matter of practical convenience because without it, the medical professionals who are at the core of the operationalisation of the idea have refused to participate. They want the "blessings" of the HPAZ and PCZ. **[20160824_My Founding Affidavit to High Court].**

Thereafter, in written correspondence to regulators and in face-to-face presentations to their decision-making committees, I emphasised the legality of the MIS and how it would operate without contravening advertising laws. E.g.

Section 135 of the Health Professions Act [Chapter 27:19], prohibits health practitioners from advertising outside the regulations of their respective professions. Subsection 135(1) of the same, defines advertising as publishing any statement or claim in a newspaper, magazine, notice, handbill, pamphlet, card or circular, and broadcasting any statement by electronic or other means except in such manner as may be specified in any regulations or rules made under this Act which define ethical practice or discipline in the health profession concerned. By these two definitions, what the MIS and all the providers who subscribe to it intend to do is therefore not advertising. The MIS will not publish any claims in print form or broadcast messages to the general public by any means. What the MIS will do is respond to individual queries or questions presented to it by patients. That is after all, the mandate of health professionals; to answer questions presented to them by patients to the best of their abilities **[20150802_MIS Letter to Health Professions Authority].**

In my view, again, on its own, similar to compliance, communication did not result in regulators endorsing the MIS. Even the framing of MIS as constitutional and legal, (the same argument presented months later by court judges), was not sufficient to change regulators’ position. it is reasoned that the arguments I presented were not effective because as observed by Maguire [41], I lacked the coercive power to compel dominant actors (i.e. the regulators) to abandon their institutionalised interpretation of advertising laws.

3. Production of texts that problematised and questioned the appropriateness of the status quo

What led regulators to conclude that what MIS proposed to do would be illegal had its basis in the principle of regulating direct-to-consumer advertising of medicines in the public interest [42] (state technical logic).

Healthcare professionals are bound by the convention that they should refrain from advertising since patients (and their families) experiencing health concerns are particularly vulnerable to persuasive emotive advertising and publicity **[20170322_HPAZ_Heads_Of_Argument].**

(N.B. Heads of Argument is a document submitted to a court of law, that summarises the key points and arguments of the case).

In response to sentiments such as the one expressed above, which emphasised advertising restrictions, I produced texts that highlighted advertising restrictions’ negative impacts on access to information. In those texts, the advertising regulations were problematised as compromising swift access to medicines and patient adherence to prescribed treatment. This assertion was couched in the professional logic, characterised by consideration for patient care. In communication with the regulators, the advertising legal framework was problematised as being inconsistent with the values of upholding patient interests.

The tragedy of [advertising restrictions] is that, patients in an economy where no pharmacy has every registered medicine in stock all the time, patients have to move door- to-door in search of the medicines they require, compromising adherence to treatment and ultimately their health. – Author **[20150802_Letter_to_HPAZ]**

Yet again, on their own, I consider that these texts did not succeed in getting regulators to alter their position on the MIS.

I now turn my attention to the institutional work carried out by multiple actors.

### Institutional work enabled by allies within and beyond the health sector

The four types of actions that I describe here were the ones that depended on the collaborative actions of multiple actors rather than the isolated efforts of one. They involved:

1. construction of a new shared understanding of medical advertising
2. litigation
3. defining/redefining concepts and ideas
4. the emergence of other actors who embraced the newly-legitimised practice of providing patients with medicine availability information

1. Allies helped with the construction of a new shared understanding of medical advertising

Before the courts were approached, my legal counsel, a human rights lawyer working *pro bono*, wrote to the regulators, explaining how the medical advertising laws “must be interpreted in the light of the Constitution of Zimbabwe” **[20160622_My_Lawyers_first_letter_to_PCZ],** i.e. in a way that promotes citizens’ constitutional rights to information and healthcare and freedom of expression.

2. Litigation

After unsuccessful attempts to secure the desired state approval from healthcare regulators, I initiated legal proceedings. I elected to take the litigation route in order to secure a different (hopefully more favourable) interpretation of advertising rules from a state authority that unlike me, possessed the coercive power to invalidate the healthcare regulators’ interpretation of advertising laws i.e. the judiciary. My decision to approach the courts was also motivated by the belief that an arbiter who was a lay person (i.e. not a health professional or healthcare regulator) would immediately find the MIS concept innovative and unproblematic, especially if they had previously faced challenges accessing medicines. I was relying on court judges considering the matter through the lens of their own [emotive] personal experience, rather than the lens of [dispassionate] regulation. The texts my legal counsel submitted to the High Court, problematised the regulators’ interpretation of the advertising rules as not only incorrect, but unconstitutional as it had the effect of potentially depriving citizens access to information and healthcare, both of which were constitutionally guaranteed rights. The same texts requested a declaration that the proposed intervention didn’t *‘contravene Section 135 of the Health Professions Act, read together with the Constitution’.* In other words, an invalidation of the institutionalised interpretation of advertising laws was sought. Litigation was enabled by the **state judicial logic**, which is preoccupied with the supremacy of constitutionalism, and which considers courts as legitimate forums, lawyers as legitimate experts and judges as legitimate decision-makers on matters, including matters concerning healthcare practice and digital technologies.

When I instigated litigation, I did not anticipate a difficult battle—a manifestation of the naïve optimism I earlier alluded to. In fact, I thought the regulators in question secretly thought the MIS was a good idea but wanted a judge and not them, to be the one to take responsibility for endorsing the MIS as being legal. What ensued genuinely surprised me because the regulators, (through their legal counsel,) put up a spirited fight, opposing my court application. Even following the High Court judgement, they appealed to the Supreme Court. Yet, I still believed the MIS was a good idea and believed that it should be obvious. Because I thought the merits of MIS were obvious, I began to entertain the belief that the MIS, the innovation, was being impeded as an attack on me personally, the innovator. With this belief came a feeling of indignation. This belief was reinforced by sentiments such as the one below, expressed in interviews I conducted with pharmacists in the study context. Similar sentiments alluded to professional jealousy being prevalent among pharmacy practitioners. In the Discussion, I reflect on the implications of this shift from optimism to indignation.

The society we have is not a society in which everyone is happy with what is happening. Do you know that someone may actually be upset that I have everything **[Interviewee describing instances of professional jealousy in the pharmacy profession]**

They[committee members] said it was a good idea but we cannot be seen endorsing this person, which was a bit strange **[Interviewee privy to debates around MIS in a certain professional association]**

A High Court ruling held that the regulators’ interpretation of advertising rules that had led them to consider MIS’ proposal illegal, was erroneous. The regulators appealed this decision to the Supreme Court, arguing that the High Court had erred in its interpretation of the medical advertising laws. Yet again, it was decided by the Supreme Court that MIS’ proposal to digitally avail medicine availability information was not in contravention of advertising regulations:

the proposed database…doesn’t contravene Section 135 of the Health Professions Act **[20181109_Supreme_Court_Order]**

(N.B. Section 135 of the Health Professions Act was the clause that prohibited direct-to-consumer medical advertising).

3. (Re)defining concepts and ideas

In delivering a court verdict that invalidated the regulators’ inhibiting interpretation of advertising rules, the High Court defined/redefined four ideas and concepts:

- the constitutional duty of state agencies (e.g. courts and regulators),
- the correct method of interpreting legislation (including advertising legislation),
- what ‘advertising’ means and
- what ‘a health institution’ means

(Re)defining these four allowed the Court to justify the invalidation of the regulators’ institutionalised interpretation of advertising laws that had inhibited them from approving MIS and its intended operations. Below, I elaborate on these four.

First, a definition of the constitutionally-enshrined duty of state agencies (including regulatory bodies and courts) was provided, before concluding that regulators had neglected that duty.

The courts and agencies of the government have a duty to ensure that fundamental rights and freedoms are protected. They must facilitate rapid and equitable development and in particular take measures to promote private initiative and self-reliance. The regulators failed to discharge their constitutional duty to consider the rights and freedoms of the applicant [Author] and those of the public, while they were considering the MIS proposal…It is a fact that they [regulators] seemed to be ignorant of such a duty.. I have no desire to emasculate the Constitution... the duty owed by the courts to the public, to assist in bringing about rapid development in the promotion of private initiative and self-reliance, enjoin me to grant the court order sought. **[20171213_High_Court_Judgement]**

In this way the court defined itself as a government entity that could remedy the regulators’ neglect of constitutional duty.

Second, the High court defined how legislation (including advertising legislation) ought to be interpreted:

An interpretation of legislation where it occurs in the interests of the public, must yield to upholding the rights enshrined in the Declaration of Rights. If the interpretation made by a tribunal discounts the guaranteed rights of an individual or the populous, then the determination will be deemed unconstitutional…Thus, because the regulators determined the MIS proposal based purely on their interpretation of the various statues without having examined the Declaration of Rights or at least referring to it, they clearly erred. **[20171213_High_Court_Judgement]**.

By defining what was the right method of interpreting legislation (the method that used a constitutionalist or rights-based lens), the High court succeeded in justifying the invalidation of the regulators’ interpretation of advertising laws that was in its view, unconstitutional.

Third, the High Court (re)defined what advertising meant in its view. Thereafter it concluded that what the MIS intended to do did not count as advertising:

the [regulator], objected to MIS on the basis that the proposed intervention was unlawful because it amounted to advertising, which is prohibited by Section 135 of the Act. To my mind, what applicant [Author] has in mind is making a publication of information by electronic means and that is most certainly NOT advertising in the sense of pitting one institution or profession against the other. **[20171213_High_Court_Judgement]**

Fourth, the High Court judge (re)defined the MIS as a health institution, contrary to the regulators who had ruled otherwise. While the regulators argued that they used the definition of a health institution provided in the Health Professions Act, the Court used the common dictionary definition of a health institution:

MIS qualifies to be described as an institution because it is ‘an organisation with a special purpose’. (Merriam Webster) dictionary)…instead of recognising that its raison de etré is that of registering health institutions and to promote the enhancement of health services for the benefit of the public, the regulator made its decision based on a restrictive interpretation of what the law deems to be a health institution **[20171213_High_Court_Judgement].**

This [re]-defining of MIS as a health institution rendered its operations (providing information) eligible to be identified as a legitimate health service, rather than acts of illegal advertising.

Both the actions of a single actor and the collaborative actions of multiple actors (including those beyond the health sector) were instrumental in challenging the interpretation of advertising laws in Zimbabwe that were effectively a barrier to a potentially useful digital health intervention. It would appear that in terms of observable effect, litigation proved to be more effective in undermining the institution in question than other discursive institutional work actions that preceded it. However, it was a record of those preceding actions convinced the High Court that other remedies had been pursued prior to approaching the Court. It was vital to prove that other actions had been tried, because when operating within the **state judiciary logic**, exhaustion of non-judicial remedies is required, before actors pursue administrative justice through Zimbabwean courts [43].

The HPAZ has insinuated that the applicant ought to have taken the matter up on appeal to the [HPAZ]…instead of approaching this Court. ….My view is that this is without merit. Appealing to the regulator is of no use to the applicant since the [HPAZ] has already stated its position. **[20171213_High_Court_Judgement].**

4. The emergence of other actors who embraced the newly-legitimised practice of providing patients with medicine availability information

Part of successfully disrupting institutions may include the adoption by other actors, of newly-emerging trends after they have been demonstrated as viable by other actors [16]. To some extent, this too was what I observed in this case study. By 2020, several interventions enabling access to medicines availability information had come into use in Zimbabwe [44–46] including a pilot run of the MIS concept. Further empirical work is required to test whether this invalidation of the institution translates into sustained change in shared meaning and norms regarding medical advertising in general and digital medicine information platforms in particular, or whether it is an uncomfortable order imposed on actors by the judiciary.

Moreover, in September 2021 I was awarded seed funding from the government of Zimbabwe to implement the MIS. The funding was awarded after a nationwide digital innovation competition run by the regulator of postal and telecommunications in Zimbabwe [47]. Receiving this funding from the government after six years of advocacy, I felt validated.

## Discussion

### Principal findings and implications for digital health system transformation

In summary, the main results showcased how the state regulators’ interpretation impeded the adoption of a potentially useful digital information-sharing intervention until an alternative, less inhibiting interpretation was validated by a more powerful state actor (the judiciary). This observation highlights the virtues of separation of powers in the governance of health systems. The institutionalised interpretation of legal provisions by powerful state actors can be a critical factor that influences if digital health interventions are adopted. Some interpretations are less enabling than others. The results also showed the possibility of change that is inherent in health systems, despite what would seem like initial resistance to digital innovations and interventions. Courses of action to bring about the desired change however, may depend on the proper identification of the {less visible] underlying cause of the resistance, which may be institutionalised ideas about how things ought to be. It is therefore worthwhile for actors involved in the implementation of digital health projects, to not focus merely on apparent barriers to implementation, e.g. the withholding of state approval. Instead, they ought to identify and focus on the less visible institutions that underlie such barriers, where they exist, e.g. the inhibiting interpretation of regulations, that ultimately precludes approval. Some authors that describe successful digital health system implementation identified the efforts of “champions” or team leaders that facilitate change management [11, 48, 49]. However, the actual actions that these champions engage in, are not described in sufficient detail to enable replication or theoretical explanation. It may well be that the activities that these ‘champions’ engage in, constitute institutional work. Having identified problematic institutions, is also worthwhile for practitioners to familiarise themselves with the forms of institutional work that are at their disposal, such as institutional logics (rule systems) in a given context, in order to effectively surmount institutionalised barriers. Health systems are particularly fertile fields for institutional work because of the plurality of logics involved.

The multiplicity of institutional logics within one context can be a useful resource for institutional workers [50] trying to surmount institutionalised barriers to digital health technologies adoption but lacking coercive power. This is because they can then take advantage of the variety. They can enlist principles from different logics to frame and seek legitimacy for their propositions. They can also engage in ‘forum-shopping’ to find one able to deliver the most favourable outcome e.g. courts (where possible; challenging state authority or other enforcers of institutions generally using litigation, may not be feasible in all settings).

It is worth noting that the invalidation of institution-based barriers to digital health interventions can come about not only due to the efforts of a leading instigating actor, but through the collective efforts of parties native to both the healthcare profession and professions beyond it e.g. members of the legal profession and digital health start-up founders who are willing to be galvanised do at least one of three things:

- Assist with intellectual and financial resources to make the institutional work of the instigating actor possible (e.g. offering pro bono legal services)
- Use their coercive power to directly invalidate the institution in question (courts)
- Implement the change once an institution has been invalidated [44–46].

This finding is consistent with some perspectives on institutional work that acknowledge the relevance of lone actors in institutional work but also concede that such work is made possible by the collective action of others [51]. More importantly, this finding further highlights the virtues of multi-sectoral working in general. Such multi-sectoral working increases the number of possible actions and legitimate decision-making forums through which positive change can be instigated (because while some actors are unable to perform certain actions because of the dominant logics that shape their behaviour, other actors can very legitimately perform those actions. For example, judges, the legitimate decision-makers in courts of law, can make decisions that coerce state regulators to adopt certain positions that impact health systems. Health professionals on the other hand, bound by the institutional logic that guides them towards complying with their regulators’ demands, are unable to similarly coerce regulators).

That all being said, it is important to highlight that while I present these recommendations for actors desiring digital health transformation, it may not be prudent to deploy them indiscriminately. Many digital health transformation projects fail to deliver on the promise of better health and economic outcomes in the contexts they are deployed because of “a too strong infatuation with technology and incapacity of formulating a clear value proposition… [52]” History has seen large investments in national digital transformation projects before anticipated benefits had been empirically verified [53]. In the section below, I reflect in particular on the potential dangers of imposing health system reform using coercive tools such as a state’s judicial apparatus.

### The paradox of judicialization in health systems reform

Finally, this case study highlights the paradox of judicialization in health systems reform. On one hand, invoking the judiciary and constitutionalism to achieve the goal of improving access to medicines can be efficacious because it results in coercive orders being issued by the courts which compel some actors to do specified actions to ensure medicine access (e.g. [54]). The case justifies recent calls for more deliberate explorations within global health scholarship and practice, of synergies between law and health [55]. The judiciary has a real potential to enable digital transformation in the health systems of developing countries, where legal challenges are some of the reasons digital health projects fail to scale [56]. On the other hand, enlisting the judiciary can have unintended negative results, such as the endorsement of a digital intervention that has not paid adequate attention to other health system objectives. Other negative results of judicialization are cited by other authors (see review by Vargas-Pelez et al. [57]) and include the undermining of public policies established through legislation, which may be more beneficial than those resulting from judicial orders. The negative consequences of judicialization of healthcare access may even widen inequalities by focusing on the needs of those able to muster legal resources for their ends.

Early in the results section, I indicated that apart from perceiving MIS’ activities as constituting illegal advertising of medicines, the HPAZ also objected to endorsing the MIS because there were no sufficient public protection mechanisms. Because there was no elaboration, it was unclear to me what this statement meant. However, following time spent undertaking academic research, systematically reviewing the literature on digital health interventions especially in developing countries, interviewing Zimbabwean pharmacists, observing their information sharing habits [21], and reflection, I appreciate the importance of this concern raised by HPAZ in its decision letter. Although the HPAZ and the PCZ did not inform me of this I came to the realization, that the MIS proposal that I submitted to them in 2015, (described earlier in this paper) had at least four deficiencies:

1. It failed to consider that some licenced pharmacies stock medicines of unverified quality [21, 58]. The MIS proposal did not include a *patient safety* plan to ensure that the pharmacies that patients accessed as a consequence of consulting the MIS digital search platform, would dispense quality-assured medicines. The issue of liability and accountability in the hypothetical event of adverse outcomes being experienced by patients who would have used the MIS platform, was also not attended to.
2. The MIS proposal did not include a plan to verify the *information* fed into the proposed MIS platform by pharmacists. A verification mechanism would enable to some extent, the protection of patients from acting on false information.
3. Also missing from the MIS proposal, was a strategy for how the MIS planned to avoid exacerbating existing *health inequalities* in Zimbabwe. Providing a digital service that only citizens with internet access and sufficient health literacy can use, is perfectly aligned with the capitalist logic of the marketplace but has the potential to widen existing inequalities [59].
4. The application of digital information management tools to healthcare-related processes unsurprisingly raises data privacy and security concerns [2, 12]. Yet, the MIS proposal lacked sufficient detail about how *data privacy and data security* issues would be handled.

I (and the courts) made little of other health system and good governance imperatives like equity, safety, accountability and data protection. The courts, enacting the values of constitutionalism inherent within the state judicial logic, similarly were inattentive to the above-mentioned deficiencies. This coincides with Bergallo’s observation [60] that courts of law tend to make decisions on healthcare interventions without considering dimensions like intervention quality and intervention effectiveness. I therefore recommend a balanced approach, which entails the judiciary remaining a responsive co-facilitator of the digital transformation of developing country health systems, while being inquisitive about and attentive to the potential implications of the digital health interventions it evaluates.

### Post-analysis reflections

The literature alludes to autoethnography being a cathartic or therapeutic research method, [26] but I was surprised to discover that it had happened to me as well. Following analysis, I had an unplanned opportunity to reflect on the effect that doing autoethnography had had on me when one of my LinkedIn primary contacts shared a public autoethnography-related post. I commented as follows on the post:

[I think] Autoethnography is the most underrated qualitative research approach that exists. It not only illuminates truths, it is therapeutic. When I did an autoethnography last year, I had to re-read certain documents that were part of the data, and I re-felt the indignation I felt when those documents were produced circa 2015/16. But at the end of the study, I felt lighter and less emotional about it all. I even managed to empathize with some of the actors that had initially inspired the indignation. That surprised me. **[July 2021_Reflections on a public LinkedIn post. Consent to share this was granted]**.

In the results section, I mentioned how I went from exhibiting naïve optimism to having feelings of indignation as I entertained the idea that MIS was being opposed as a personal attack. Reflecting on what the outcome of this change was, I realised that it had both advantages and downsides. On one hand, the indignation fuelled my aggressiveness and desire to triumph and implement the MIS despite the challenge. On the other hand, I think the indignation made me less perceptive to the genuine shortcomings of the MIS proposal. Because I was suddenly hypervigilant against opposition, I was vulnerable to dismissing genuine critiques of the MIS proposal as personal attacks. I write this as a cautionary example to proposers and reviewers of new digital innovations, interventions or policies in the healthcare space. I strongly recommend that policy debates must not turn into or be seen to be turning into personal battles. It can then undermine opportunities for the kind of dialogue that can improve the intervention overall.

### Limitations

The findings of this study must be read alongside the limitations of the methods employed. The limitations of autoethnographic work and how their impacts were mitigated in this study through peer review, member-checking and triangulation have already been discussed in the Methods. A second limitation was the lack of access to the internal minutes of meetings held by regulatory officers as they considered the MIS proposal. I only had access to their correspondence to me or my legal counsel. Representatives of the regulatory institutions were however interviewed both formally and informally [21]. I consulted one of them consulted on the contents of this paper.

## Conclusion

The barriers to the uptake of digital health systems can be linked to underlying institutions or institutionalised interpretations of reality. Attempting to challenge the barrier without addressing the underpinning institution may not yield desired results. Institutionalised barriers are especially problematic because they resist change and are often not identifiable as such, before institutional analysis. These institutions can be especially potent if they are perpetuated by powerful actors capable of enforcing compliance through legal frameworks. This study sought to explain how actors can overcome such institutionalised barriers to digital health system implementation. After identifying a barrier as being institutionalised or linked to an institution, actors might challenge such barriers by engaging in institutional work; i.e. deliberate efforts to challenge the relevant institution (e.g. a law, norm or shared belief). Institutional work may require the actions of multiple actors within and beyond the health sector, including judicial actors. Such cross-sectoral alliances are efficacious because they provide institutional workers with a broader range of strategies, framings, concepts and forums with which to challenge institutionalised barriers. However, allies beyond the health system (e.g. the judiciary) must be inquisitive about and attentive to the health system implications of the digital health interventions they champion.

## Data Availability

Excerpts from the data that support the interpretations are contained in this published article as quotes. The complete datasets (that includes interview transcripts) used and/or analysed during the current study are available from the corresponding author on reasonable request.

## Abbreviations

HPAZ: Health Professions Authority of Zimbabwe
MIS: The Medical Information Service
PCZ: Pharmacists Council of Zimbabwe
WHO: World Health Organization
